# Genetic Mapping for Agronomic Traits in IAPAR 81/LP97-28 Population of Common Bean (*Phaseolus vulgaris* L.) under Drought Conditions

**DOI:** 10.3390/plants10081568

**Published:** 2021-07-30

**Authors:** Júlio César Ferreira Elias, Maria Celeste Gonçalves-Vidigal, Mariana Vaz Bisneta, Giseli Valentini, Pedro Soares Vidigal Filho, Thiago Alexandre Santana Gilio, Vânia Moda-Cirino, Qijian Song

**Affiliations:** 1 Departamento de Agronomia, Universidade Estadual de Maringá—UEM, Av. Colombo 5790, Maringá 87020-900, PR, Brazil; juliocesar_net@hotmail.com (J.C.F.E.); marianavazbisneta@hotmail.com (M.V.B.); psvfilho@uem.br (P.S.V.F.); 2 Soybean Genomics and Improvement Laboratory, US Department of Agriculture, Agricultural Research Service (USDA-ARS), Beltsville, MD 20705, USA; Giseli.Valentini@usda.gov (G.V.); qijian.song@usda.gov (Q.S.); 3 Programa de Pós-graduação em Genética e Melhoramento de Plantas, Universidade do Estado de Mato Grosso, Cáceres 78217-900, MT, Brazil; thiago_gilio@hotmail.com; 4 Instituto Agronômico do Paraná—IAPAR, Rua Celso Garcia Cid, km 375, Londrina 86047-902, PR, Brazil; vamoci@iapar.br

**Keywords:** abiotic stress, QTL-quantitative trait loci, *Phaseolus vulgaris* L., water deficit

## Abstract

One of the significant challenges of common bean breeding is developing cultivars with high yields under drought conditions. The present study attempted to map quantitative trait loci (QTLs) and identify molecular markers that are linked to drought tolerance in the common bean. We evaluated 160 recombinant inbred lines (RILs), derived from the cross between the carioca cultivars IAPAR 81 (drought tolerant) and LP97-28 (susceptible to drought). In 2014 and 2015, two experiments were conducted (DS—drought stress, and NS—no drought stress). In the DS experiment, water suppression was performed at the flowering stages R5 to R6. The results of our experiments showed that drought conditions play an essential role in reducing most of the traits that were evaluated. RILs under drought conditions reduced the grain yield by 62.03% and 24% in 2014 and 2015, respectively. We identified 15 quantitative trait loci distributed on the chromosomes Pv01, Pv02, Pv03, Pv07, Pv08, Pv09, Pv10, and Pv11, related to grain yield, seed yield per day, 100-seed weight, number of pods per plant, plant height, number of days for flowering, and number of days to maturity. The characteristics of seed yield per day, 100-seed weight, and number of days to maturity showed that QTLs colocalized on Pv07. Identifying QTLs that are linked to drought tolerance in the RIL population IAPAR 81 × LP97-28 is of particular importance for common bean breeding programs seeking to improve carioca beans that are cultivated in regions with drought conditions, such as Brazil.

## 1. Introduction

The common bean is an essential legume for the human diet, as it represents a vital source of nutrition and income for people, especially those from African and Latin American countries [1,2,3,4]. The common bean is considered to be a subsistence crop in several regions globally, especially regions that are characterized by soil with low-quality, adequate fertilization, and susceptibility to biotic and abiotic stresses. Among these stresses, a lack of rain is the primary cause of the low productivity of the common bean, since the cultivation of the mentioned legume relies mainly on rainfall.

Water deficit in the common bean (*Phaseolus vulgaris* L.) causes severe restrictions to productivity, which can directly affect the morphophysiology of the crop during any cycle stage. The incidence of short-term drought periods in the main common bean-producing regions, such as Brazil, Mexico, the United States, and African countries, tends to increase due to climate change, which negatively impacts global production and food security [5,6,7]. According to Diaz [2], drought represents a high-risk factor for bean production, and improved varieties that withstand the altered climatic conditions constitute a valuable resource. Therefore, efforts are necessary to improve the crop tolerance to stress conditions, particularly drought, to reduce poverty and malnutrition in developing countries in tropical regions [8].

One of the most significant breeding program challenges is developing cultivars with a substantial production capacity under water stress conditions [9]. In this context, plant breeding that is focused on drought tolerance offers a useful, efficient, and enduring method to ensure sustainable common bean productivity, even under unfavorable water-deficit conditions [8,10].

Brazil is the third largest global producer, and one of the largest consumer markets, of grains from the *Phaseolus* genus, characterized either by subsistence or mechanized agriculture. In both the scenarios, abiotic factors (e.g., high aluminum toxicity, sandy soil with low nutrients, and drought) can restrict bean cultivation, and consequently reduce bean yield [11].

Common bean productivity in Brazil can vary according to the season, and this is closely related to the rain supply. During rainy-season production, sowing occurs in the months that show a high frequency of rain, with the harvest occurring between November and February. Dry-season production, with irrigation supplementation, is concentrated in some regions of Brazil, mainly for the central region, where highly technical cropping systems and central-pivot irrigation have been adopted. Winter, or third-season, production takes place from May to September. This scenario shows that beans can be grown year round, guaranteeing the permanent supply of this product on the market; however, the average production is highly unstable, and varies according to the growing region [12].

The common beans of Mesoamerican origin are the most cultivated in Brazil [13], because of the consumer-preferred grains of black and carioca commercial types. The vast diversity that is present in Mesoamerican and Andean genotypes provides levels of drought tolerance. Consequently, common bean breeders can profit from these germplasms, to develop drought-tolerant cultivars [8,14].

Mesoamerica and Durango races have been frequently described as drought-tolerance sources to develop cultivars for environments that are affected by abiotic stress [15,16,17]. Despite that, studies have revealed little progress in the transference of drought-tolerant genes to commercial cultivars, through hybridization between the two bean gene pools [18,19].

Biomass accumulation in aerial parts and roots, translocation of photoassimilates to seeds, water stress harvesting, and pod harvest indices are examples of traits that are associated with grain production stability under water stress [8,20,21,22]. An optimal strategy to tolerate drought would be a deep root system with long secondary and tertiary roots that allow for water extraction from the deeper soil layers [23,24,25].

The change in characteristics, such as the number of days for flowering and maturation for water supply, is an essential strategy for adapting common beans to water shortages. Hoyos-Villegas et al. [26] reported that water conservation might limit photosynthesis, and the growth and development of plants. In response to severe drought stress, bean plants may produce smaller or fewer leaves, resulting in suboptimal leaf area and reduced net photosynthesis. Schneider et al. [27] suggested selection for increased biomass under drought stress in order to avoid a reduction in photosynthetic capacity. This practice, however, may result in indirect selection for later maturity.

Beebe et al. [1] noted that mechanisms to escape drought include early maturity, phenotypic plasticity, and rapid partitioning of photosynthates to seeds. Selection for earlier maturity may help avoid terminal drought, but early maturity may reduce the seed yield potential during more favorable growing seasons. An early, defined, and irreversible shift to reproductive development, and a shorter period of pod filling, could reduce the exposure during the sensitive reproductive period of growth, and shorten the growing season, thus increasing the chances of escape [28]. The characteristics that are related to drought tolerance are usually controlled by the genetic factors of polygenic inheritance [7,10,27]. However, further detailed studies are needed in order to better understand all the mechanisms that are involved in drought tolerance, and ease gene transference to new cultivars.

Efficient water use strategies, through root systems and crop development, are crucial factors for common bean adaptation to drought conditions [15,29]. Therefore, drought affects common beans directly, by reducing their growth and causing plant death [29]. Likewise, a plant under a high water stress index becomes more susceptible to plagues and diseases [30]. In contrast, a healthy plant, with genetic mechanisms for drought tolerance, could maintain production stability under drought conditions. The genetic control of the traits that determine the drought tolerance of the common bean may suffer variations according to the stress conditions to which the genotypes are submitted [1,31].

The investigation of the genetic control of this type of trait can be performed by genetic mapping and association analysis, aiming to identify genomic regions controlling phenotypic traits. Until recently, the common bean genotyping ability was limited, which compromised the accuracy of the QTLs that were mapped for water stress, but this situation has changed in recent years, with technological evolution in genotyping, such as the bead chip, genotyping by sequencing, reference genome, and phenotyping advances.

Traub et al. [9] evaluated the metabolic and photosynthetic responses to drought stress in *P. vulgaris* and *P.acutifolius*. Mukeshimana et al. [8] identified QTLs that were associated with the yield components under drought tolerance in an intergenic common bean population (SEA 5 × CAL 93), on chromosomes Pv03, Pv07, and Pv09. Asfaw et al. [24] identified QTLs for the characteristic yield and SPAD chlorophyll meter reading on Pv08 and Pv06, respectively, under water stress conditions. Additionally, with the Mesoamerican population from the Buster × Roza cross, significant QTLs were mapped on the chromosomes Pv01 and Pv02, for yield and phenological traits [10]. By nested association mapping (NAM), Hoyos-Villegas et al. [26] reported QTNs on Pv03 and Pv10, with effects that were associated with yield components under drought stress. Dramadri et al. [32], using an RIL population from Ecuador (drought tolerant) × Red Hawk, mapped eighteen QTLs that were associated with the phenology, yield components, and partitioning traits on the chromosomes Pv01, Pv02, Pv03, Pv04, Pv06, and Pv11. The purpose of this study was to recognize essential traits under water stress, define the genetic effects of drought stress in 160 common bean recombinant inbred lines (RILs) from cross IAPAR 81 (tolerant) × LP 97-28 (susceptible), and identify qualitative trait loci (QTLs) that are related to drought-tolerant traits in this population.

## 2. Results

### 2.1. Level of Water Stress and Its Effects on Genotype Performance

**Figure 1:**
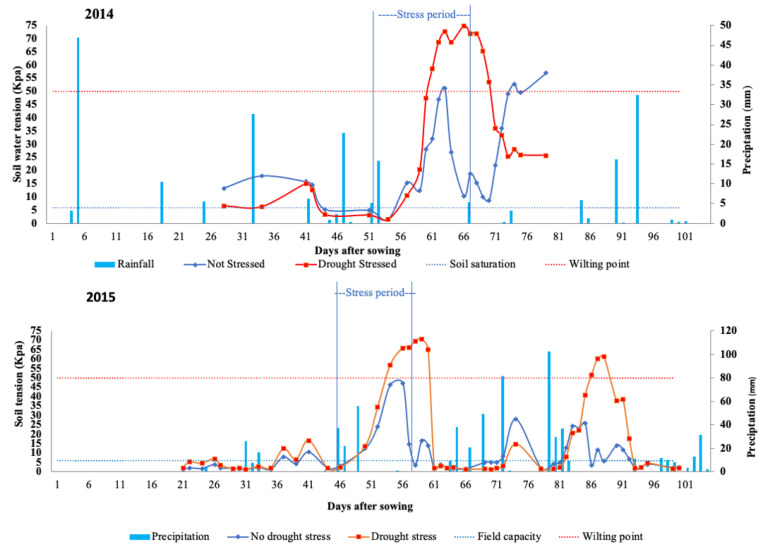
Soil water tension for drought stress (DS) and no-drought stress (NS) conditions and precipitation (mm) in the experiments conducted at the Technical Irrigation Center (CTI) in Maringá, PR, Brazil in 2014 and 2015. Dotted lines indicate the field capacity and wilt point according to the soil water tension values.

In the area where we performed the experiments, during 2014 and 2015, the precipitation was insufficient for the optimal development of common bean plants. Consequently, we implemented irrigation water management. The average temperatures were suitable for normal plant development during the same period; the average daily temperature in 2014 was 23.73 °C, whereas in 2015, it was 23.45 °C (Figure 1).

The precipitation in 2014 was 220 mm, of which 132 mm accumulated until the beginning of the flowering season (R2). Furthermore, in 2015, a total of 599 mm of accumulated rainfall was observed during the whole experimental period. Only 386 mm was observed during the maturation phase. Thus, the experiment described as DS (with drought stress), in 2014 and 2015, suffered a sporadic intraseasonal drought at the beginning of flowering, contributing to an adequate evaluation of the drought effect on the bean lines at the flowering stage.

The drought intensity index (DII) evaluates the behavior of genotypes under DS (with drought stress) and NS (no drought stress) conditions, as shown in Table 1. The drought intensity index for the grain yield was moderate, and the average value for 2014 and 2015 was DII = 0.44. The drought effect on grain yield was more severe in 2014, with DII = 0.59, whereas in 2015, this index value was 0.30. The parents exhibited mean index values of DII = 0.70 and DII = 0.38 in 2014 and 2015, respectively. However, contrasting DII values were observed for the parents in 2015; IAPAR 81 (tolerant to drought) showed a low drought index (DII = 0.21), but LP 97-28 (not susceptible to drought) underwent severe drought and exhibited a DII value of 0.50.

### 2.2. Phenotypic Data

In 2014, all the evaluated traits were significantly affected by the drought stress conditions during the flowering stage, except for the number of seeds per pod (Table 2). The average grain yields of the 160 recombinant inbred lines (RILs), under NS and DS conditions, were 2363.49 and 897.38 kg ha^−1^, respectively. The outcome was a reduction in 62.03% of the grain yield in the RILs under water drought conditions. The grain yield estimates for the parents were 3381.50 kg ha^−1^ for IAPAR 81, and 2048.17 kg ha^−1^ for LP 97-28, under no-drought stress conditions (NS). Under DS conditions, the IAPAR 81 grain yield was 16.39% higher than LP 97-28 (IAPAR 81 GY = 980.23 kg ha^−1^ and LP 97-28 GY = 819.52 kg ha^−1^). The weights of 100 seeds of RILs were 17.79 g and 19.39 g for the DS and NS conditions, respectively.

The SW for the parents presented intermediate values of those shown by the RILs. The SW ranged from 16.77 to 19.70 g in the DS condition, and it varied from 17.84 to 25.68 g in the NS condition, demonstrating that water deficit enhanced its reduction. The RILs exhibited an average number of 11.41 pods per plant and 5.60 seeds per pod under the NS condition, while these values were 8.03 pods and 5.13 seeds under the DS condition. There were no observed differences in the number of days for flowering on the parents and RILs under the NS and DS conditions. However, genotypes that were cultivated under drought conditions (DS) showed a reduced interval for grain filling and maturity than those that were cultivated under non-drought conditions (NS).

In 2015, highly significant differences were observed between the study conditions DS and NS, for GY, NP, SW, PH, and NDF (Table 2). Moreover, genotype-by-environment condition interactions (G × E) exhibited significant differences for all the evaluated characteristics. We observed a decline in all the traits for both the experimental treatments during the agricultural year of 2015. GY for the RILs ranged from 744.72 to 1508.50 kg ha^−1^ under the NS conditions, whereas this trait varied from 521.96 to 1621.87 kg ha^−1^ under the DS conditions. Exposure to drought stress caused an average reduction in GY and SYD of 24% and 30%, respectively. SW varied from 19.60 to 23.78 in the NS treatment, while the interval for DS treatment ranged from 18.97 to 22.68 g. The mean NP was 8.61 (NS) and 7.23 (DS), whereas the average PH values for the NS and DS experiments were 50.49 and 45.65 cm, respectively. NDF, NDM, and NDE showed the highest mean values throughout the year compared to the previous experimental year (2014).

In both the years (2014 and 2015), the RILs showed higher values than the parents for the grain yield and yield components, which characterized the presence of transgressive segregation in this population (Figure 1). Additionally, the normal distribution of all the evaluated traits shows that genetic control is inherited quantitatively.

**Figure 2:**
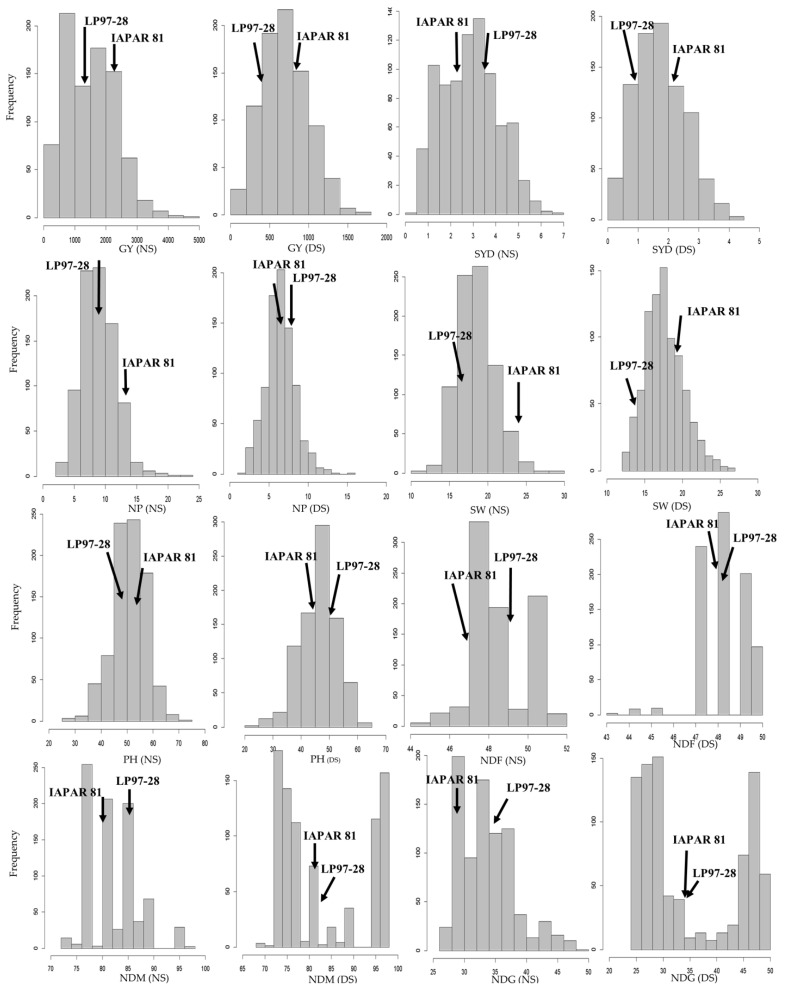
Distributions of the frequencies for the traits evaluated in the field: GY = grain yield; SYD = seed yield per day; NP = number of pods per plant; SW = 100-seed weight; PH = plant height; NDF = number of days for flowering; NDM = number of days to mature; and NDG = number of days to grain filling for the population of recombinant lines (RIL) from IAPAR 81 × LP97-28 cross under drought stress (DS) and no-drought stress (NS) conditions in Maringá, PR, Brazil in the years 2014 and 2015.

The phenotypic correlation coefficients for GY and other variables were higher in the NS experiment than in the DS experiment (Table 3). Positive correlations between SYD and the yield components have previously been reported under drought conditions [8,33,34,35]. The GY was positively correlated with SYD (r = 0.73), NP (r = 0.65), and SW (r = 0.42). The correlation between NP and SYD was positive and significant (r = 0.57). Moreover, the 100-seed weight and SYD revealed a positive and significant correlation (r = 0.35). Under the DS condition, the correlation was positive and significant between the yield components for NP and SYD (r = 0.72), and SW and SYD (r = 0.46). The correlations among NDE and NDM were positive and highly significant in the DS (r = 0.93) and NS (r = 0.84) experimental conditions. Conversely, the grain yield and its primary components and phenology traits showed negative correlations(Figure 2).

### 2.3. Genetic Mapping

This study genotyped 160 RILs from the IAPAR 81 × LP97-28 cross with 5.398 SNPs. A total of 1.287 SNPs were polymorphic between the parents. After excluding the markers that did not meet the minimum allele frequency of 30%, we selected a total of 773 SNP markers for mapping procedures. Mapping of the IAPAR81 × LP97-28 population covered 815.8 cM, with an average distance between the markers of 1.34 cM (Table 4). The number of markers per chromosome ranged from 23 (Pv04 and Pv11) to 195 in Pv02. The chromosome Pv04 had the shortest length, at 13.1 cM, while Pv01 had the longest length, at 117.9 cM.

### 2.4. QTLs Mapping

**Figure 3:**
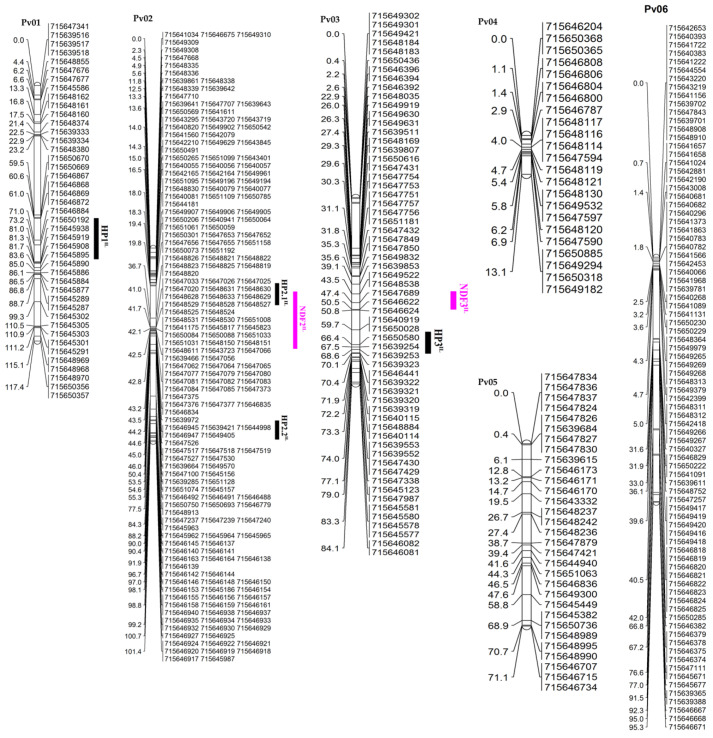

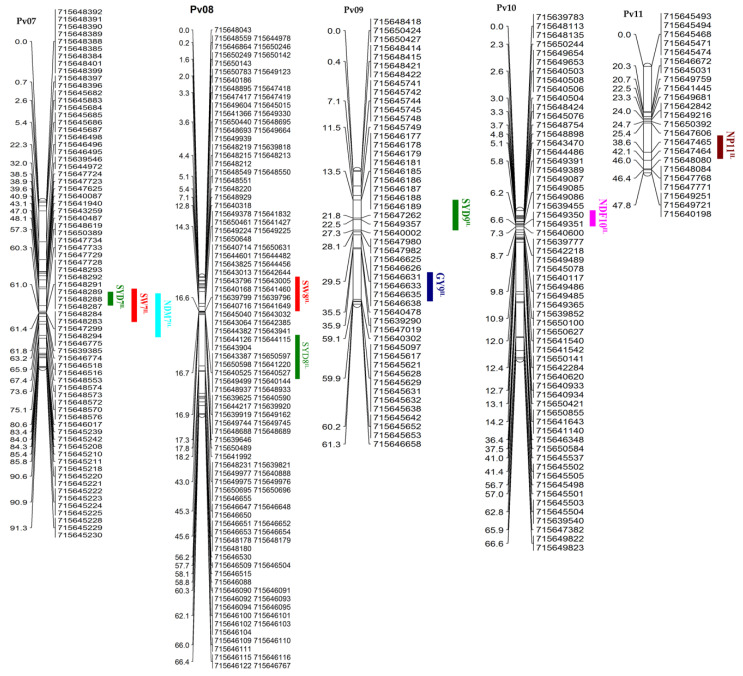
Genetic map for the common bean RIL population from IAPAR 81 × LP97-28 cross using 773 SNPs markers assigned to the 11 common bean chromosomes. QTLs mapped for each trait are colored as follows: GY = grain yield in dark blue; SYD = seed yield per day in green; NP = number of pods per plant in brown; SW = 100-seed weight in red; PH = plant height in black; NDF = number of days for flowering in pink; NDM = number of days to mature in light blue.

A total of 15 QTLs, associated with GY, SYD, SW, NP, PH, NDF, and NDM, were identified on Pv01, Pv02, Pv03, Pv07, Pv08, Pv09, Pv10, and Pv11 (Table 5, Figure 3). The QTLs that were associated with the GY (NS) conditions were mapped in Pv09, linked to the marker ss715640302, in the interval of 46.7 to 59.9 cM, and accounted for 8.7% of the phenotypic variance. SYD exhibited QTLs mapped on the chromosomes Pv07, Pv08, and Pv09. Additionally, QTLs in Pv07 in 2014, under drought stress, were linked to the ss715640487 marker, within a mapping interval from 49.8 to 57.1 cM. For the combined analysis (2014 and 2015), under drought stress, we identified QTLs that were mapped on Pv08 in 2014 and 2015, and linked to the marker ss715648231, with a peak position of 43.0 cM. The QTL in Pv09 was mapped between 13.5 and 27.3 cM to the ss715649357 marker, under NS conditions, in 2014. SYD suggests the presence of alleles that were originally from IAPAR81, under both the experimental conditions DS and NS. These QTLs demonstrated phenotypic variations of 12.1, 9.0, and 8.3%.

Figure 3 shows the order and location of the SNP marker in the linkage groups that agreed with the ranking and assignment of chromosomes on the common bean map from the Stampede/Red Hawk RIL population [36].

#### 2.4.1. QTLs for 100-Seed Weight (SW)

Interestingly, we observed that the QTLs on Pv07 and Pv08, controlling SW in the population, were derived from the cross IAPAR81 × LP97-28. On Pv07, QTL is linked to the marker ss715647728 at 61 cM, for experiments DS and NS, and the combination of years.

In 2014, this QTL was identified under the DS condition linked to the SNP marker ss715639385. Likewise, the QTLs for SW on Pv08, which were inherited from the parental LP97-28, were found under NS and DS conditions. The most common SNP markers linked to this QTL were ss715648929, ss715648219, and ss715648220. The QTLs for SW showed a more constant behavior across the experimental years in comparison to other agricultural traits, such as SYD, NP, and PH (Table 5).

#### 2.4.2. QTLs for Number of Pods per Plant (NP)

QTL mapping for the number of pods per plant showed stable performance across the experimental years. Beneficial alleles, derived from IAPAR81, were expressed under DS conditions, whereas negative alleles, donated from LP97-28, were expressed under DS conditions. One QTL was found in Pv11 under the DS condition, in both the experimental years. This QTL is linked to the marker ss715647465 at intervals of 31.1 and 41.4 cM, and contributes 13% of the phenotypic variation (Table 5).

#### 2.4.3. QTLs for Plant Height (PH)

Three QTLs for PH were mapped in Pv01, Pv02, and Pv03, under DS conditions for each year and the combined analysis (both the experimental conditions). The QTL in Pv01 linked to ss715646868 marked at 67.0 cM for both the years and combined research under DS conditions. LP97-28 drought tolerance alleles contributed to PH, and accounted for 8.9% of the phenotypic variation. Two QTLs were found on Pv02, the first being in the NS condition, located in the interval of 18.5 to 29.8 cM, with a phenotypic variation of 8.7%, and is linked to the marker ss715640941. The second QTL was found in both the years and the combined analysis, mainly under drought conditions. The markers ss715646144 and ss715646929 were linked to the second QTL, explaining a phenotypic variation of approximately 9%. Only a single QTL for PH (under DS conditions) was identified in Pv03, through combined analysis across the years. Beneficial alleles (donated from IAPAR81) were linked to the marker ss715650580, and explained 9.5% of the phenotypic variation (Table 5).

#### 2.4.4. QTLs for Number of Days to Flowering (NDF)

We detected a total of three QTLs flowering on the chromosomes Pv02, Pv03, and Pv10 for several days (Table 5). The QTL found in Pv02 in 2014, and in the combined analysis under NS conditions, explained more than 10% of the phenotypic variance, and was linked to the markers ss715648819, ss715647526, and ss715647652. In the experiment (NS) that was conducted in 2014, we found a QTL on Pv03 that was linked to the marker ss715647689, accounting for 12.3% of the phenotypic variance. The QTL (controlled by IAPAR81) was mapped in Pv10, linked to the marker ss71539783, under DS conditions in the combined analyses of the years and accounted for 8.6% of the phenotypic variation.

#### 2.4.5. QTLs for Number of Days to Maturity (NDM)

Regarding the number of days to maturity, one QTL was identified in Pv07. In the experiment DS, 2014 and NS, 2015, and for the combined analysis, we identified QTLs that were linked to five markers at intervals starting from 50.6 to 74.5 cM.

## 3. Discussion

### 3.1. Genetic Diversity

At the molecular level, recent studies based on sequence data indicate that common beans are of Mesoamerican origin and originate in Mexico [12,14]. In Brazil, there is a predominance of Mesoamerican cultivars, which present a high level of genetic diversity when compared to the Andean cultivars [7].

In the present study, the RIL population derived from the cross IAPAR 81 × LP97-28 was used to map the QTLs that were related to drought tolerance in the common bean. The two parents IAPAR 81 and LP97-28 that were involved in the cross have Mesoamerican origin and belong to the carioca market class, which is the most consumed market class in Brazil. Both the parents contrasted in several traits related to drought tolerance, such as days to flowering and guiding, seed protein content, and grain yield. Most of the QTLs and markers associated with drought in the common bean have been based on crosses between the Mesoamerican and Andean genotypes, to facilitate abundant DNA polymorphisms [33]. According to the same authors, since most genetic breeding is carried out within the gene pools or market classes, there is still a need for markers that will permit selection among genetically similar materials, such as carioca beans.

### 3.2. Phenotypic Data

The common bean requires at least 100 mm of rainfall per month for appropriate development. In 2014, the precipitation in the area where the experiments were conducted was not sufficient for the optimal development of the bean plants. The rainfall was 200 mm throughout the entire crop cycle in 2014. On the other hand, in 2015, there was sufficient precipitation (599 mm), ensuring good crop development. However, the rainfall during the experiments conducted in 2015 was not regular, providing intervals that were characterized by no rain and high temperatures.

In both 2014 and 2015, the rain pattern during the experiment led to prolonged drought in the initial stages of plant development. Thus, the IAPAR 81 × LP97-28 RIL population was exposed to a range of stresses throughout the development stage (water deficit combined with high temperatures), representing the actual conditions in Brazilian producing areas, and contributing to identifying QTLs for drought tolerance in the common bean. Common bean plants are susceptible to water stress, which, especially when combined with high temperatures, produces effects such as abortion of flowers, pods, and grains that directly affect the grain yield [1].

In general, the IAPAR 81 × LP97-28 population had a maturation period that was longer in 2015 than in 2014. In 2014, the minimum temperature was 14.82 °C, and the maximum temperature was 31.34 °C. In 2015, the minimum and maximum temperatures were more pronounced, at 17.20 and 37 °C, respectively. Several authors have described that the common bean grows faster when the temperatures are elevated, due to flower, pod, and grain abortion, directly reflecting the low grain yield [8,16]. Thus, the average yield of grains under water deficit was higher in 2014 than in 2015.

The performance of the RILs was characterized by a transgressive genetic effect on the grain yield and the grain yield components that were inherited by the drought-tolerant parent used in the cross, that is, IAPAR 81 (Figure 2). We identified transgressive segregation for the grain yield in intragenic Mesoamerican crosses. Similar results were observed in the DOR364 × BAT 477 population [7] and Yuste-Lisbona et al. [37] in the intergenic population PMB0222 × PHA1037. Beebe et al. [1] reported that the transgressive segregation of RILs is due to the complementarity and/or mechanisms of the parents that allow the expression for drought tolerance. Diaz et al. [2] studied a multiparent advanced generation intercross (MAGIC) and noticed significant genetic variability within all the traits, and transgressive segregation was in most of them.

The average 100-seed weight that was observed in the experiment for the RILs was slightly lower than the 100-seed weight from the parent IAPAR 81 under drought stress and no drought stress conditions in both the years. According to other studies, the effect of water stress shows a slight reduction in the 100-seed weight of common beans [8,10,11,16]. Grain filling is inhibited under water stress, so RILs with greater than 100-seed weight are indicated as the most tolerant to drought [20]. When the DII (stress intensity) is light, the 100-seed weight presents itself as a stable characteristic over the years, which would make it worthwhile for the drought tolerance breeding of common beans [7]. Schneider et al. [27] demonstrated that the 100-seed weight in common beans is relatively stable, with the DII ranging from 0.19 to 0.49. The fact that the 100-seed weight correlated with the number of pods per plant under both the NS and DS stress conditions in 2014 and 2015 indicates that the genes that are responsible for the grain yield are linked to the genes that control the 100-seed weight, signifying the presence of pleiotropy. This claim is further supported by identifying QTLs that are related to the 100-seed weight and yield per day, co-locating in the same link group Pv07. This co-segregation of genes for the 100-seed weight and yield per day facilitates the simultaneous selection of lines with high grain yield in this Mesoamerican population.

Nonsignificant negative correlations between phenology and grain yield characteristics in the NS condition, and significant negative correlations in the DS condition, can be explained by the rainfall variation in the experimental locations. That is, days without rain in the initial flowering stage characterize an intermittent drought instead of the terminal drought that reaches the grain ripening phase.

The characteristics of grain yield per day, number of pods per plant, and 100-seed weight showed a correlation with grain yield for the non-drought stress condition in 2014 and 2015 (Table 3). On the other hand, no association between grain yield and yield-related traits was observed under the drought stress conditions. The number of days for flowering and maturation, and the days for filling, were negatively correlated with the grain yield components under drought stress, suggesting that maturation was necessary in order to escape drought in the two agricultural years. These correlation effects indicate that, depending on the intensity of the deficit for each year, several resistance mechanisms contributed to the complete performance of this population. These effects also demonstrate the complexity of breeding for drought tolerance in the common bean, mainly to address the local stress conditions and annual variation [8].

### 3.3. QTLs Mapping

In this study, the presence of transgressive segregation had an essential effect on the populations of Mesoamerican origin, and it was very noticeable for the grain yield and yield components (Figure 2). Similar results were observed by Diaz et al. [2] for DF, DM, SW, and productivity characteristics. We identified quantitative trait loci for several characteristics that were mapped on the same chromosome region, such as those on Pv07 and Pv09. This fact may be related to the cause–effect structure, which indicates that these characteristics may be conditioned by physically linked genes (pleiotropic) [8,24]. According to the criteria of Frei et al. [38], Miklas et al. [31], Assefa et al. [39], Beebe et al. [1], and Mukeshimana et al. [8], for studies that evaluate the performance of genotypes under water stress, identical genotypes must be assessed under normal irrigation conditions and under water restriction. In the present study, we met these conditions, showing that the genotypes’ performance grew in a stressed field condition, where water supplementation was restricted at the beginning of the flowering stage, and a stress-free field condition, with good water maintenance, as required for normal plant development.

The QTL GY9^IL^ for grain yield was identified on chromosome Pv09, under NS conditions, in 2014. No association was found for grain yield under drought stress in Pv09, since the allele contribution was from the parent LP97-28 in the individual harvests and combined analyses. Several studies have described the presence of QTLs for grain yields in Pv09 [8,40]. An essential challenge for breeders working with certain classes of common beans has been the negative association between seed weight and productive potential in some crosses [8,40]. In the present study, positive correlations were observed in grain yield, concerning production components. In addition, the grain yield alleles per day (SYD7^IL^) and 100-seed weight (SW7^IL^) that were identified under the stress condition, were contributed by the parent LP97-28, consequently favoring the simultaneous selection of two characteristics in this cross between the Mesoamerican cultivars.

QTLs for the number of pods per plant trait, mapped in Pv11 (NP11^IL^), were identified under DS conditions, which indicates that this QTL has an indispensable effect on the number of pods per plant, and can be used to select beans with a higher number of pods per plant under drought-stressed conditions. Quantitative trait loci for pods per plant were previously mapped to Pv07, Pv09, and Pv11 in populations in an intergenic population with advanced backcrossing [41]. In studies conducted by Mukeshimana et al. [8], QTLs in Pv03 and Pv08 were identified. Moreover, Checa and Blair [42] mapped QTLs for the number of pods per plant on Pv04 and Pv10, although Beattie et al. [43] mapped QTLs for the number of pods per plant on Pv03. Although it is difficult to compare these QTLs, due to the different marker technologies that were used, there is strong evidence for the presence of QTLs affecting the number of pods per plant on Pv03.

Seed-weight QTLs were exclusively mapped in two genomic regions on Pv07 and Pv08. A highly expressive QTL for 100-seed weight, SW8^IL^, explained between 8.5 and 21% of the phenotypic variation. This QTL was found in 2015 and studied for two years, and combined for DS and NS conditions and under DS conditions in 2014. A second QTL, SW7IL, was consistently mapped to Pv07 under DS and NS conditions. This QTL was mapped in the same QTL regions for grain yield per day (SYD7^IL^) and the number of days for maturation (NDM7^IL^). The connection of different QTLs in Pv07 provides an option for simultaneous selection for 100-seed weight, and indirect selection for grain yield and maturation. These results agree with several previous studies that mapped QTLs to 100-seed weight in Pv07 [8,40,44]. The location of QTLs for 100-seed weight in Pv07 reinforces the link with the phaseolin protein locus in seeds (Phs), which was previously mentioned in several populations using different protein identification methodologies [42,44,45].

In this work, three (PH1^IL^, PH2^IL^, PH3^IL^) QTLs for plant height were observed on the chromosomes Pv01, Pv02, and Pv03, under DS, showing consistency between 2014 and 2015. Previous studies, conducted by Diaz et al. [46] and Asfaw et al. [24], described PHI QTLs on the chromosomes Pv06 and Pv08, and these chromosomal regions would be considered alternatives for breeding programs. Previous studies identified QTLs that were related to plant height, located on the Pv03 chromosomes, Pv10 and Pv11 [42,43,47], and in Pv6 [48].

An essential mechanism for adapting to water stress is phenological flexibility [8,22,40]. In the present study, QTLs for the number of days for flowering for the stressed condition (DS) were mapped on Pv10, corroborating the studies that were carried out by Kwak et al. [49] and Mukeshimana et al. [8]. In comparison, the QTLs for NDF under NS conditions were found in Pv02 and Pv03. The QTL for the number of maturation days located on Pv07, under DS and NS conditions, explained 8.4% to 12.1% of the phenotypic variation, respectively, constituting only the favorable alleles of the parental IAPAR81. Similar results were observed in several studies involving different populations of common beans under water stress [8,9,10,24,26,31].

DNA markers improve the efficiency and precision of plant breeding via assisted selection in the development of new common bean cultivars that are tolerant to water deficiency. Therefore, there is extensive variability in the parent under field conditions, making the selection of promising lines under field conditions a critical part of the breeding program. Using a high-resolution genetic map and phenotypic field data under different water deficit conditions, QTLs are linked to traits that guarantee the mapping of adaptation of common beans to water stress environments. Additionally, the availability of molecular markers that are linked to drought tolerance traits, which were identified through crosses between genotypes of the carioca commercial group (IAPAR81 × LP97-28), is of particular importance for common bean breeding programs in Brazil, as the commercial group carioca is the most produced and consumed.

## 4. Material and Methods

### 4.1. Population Development

In the present study, 160 recombinant inbred lines (RILs) derived from the cross between IAPAR 81 × LP 97-28 were evaluated. The F1 generation of the cross IAPAR 81 × LP 97-28 was obtained at the Instituto Agronômico do Paraná (IAPAR) (Londrina, PR, Brazil). The F2 to F6 generations were conducted through the single-seed descendant (SSD) method in a greenhouse at the Núcleo de Pesquisa Aplicada à Agricultura (Nupagri) of the Universidade Estadual de Maringá (UEM) (Maringá, PR, Brazil). The IAPAR 81 cultivar is known to be tolerant to water deficiency.

According to Moda-Cirino et al. [50], the IAPAR 81 cultivar was developed from multiple crossings (IAPAR 81 = (A248 × EM P 117) × {Araúna Selection × [BAT 93 × (Carioca Selection 99 × Great Northern Nebraska 1 Selection 27)]}) between lines considered resistant to disease and was released in July 1997 in Paraná state. This cultivar has an indeterminate growth habit, which allows mechanical harvesting. The average flowering time of IAPAR 81 was 43 days and 92 days from emergence to harvest. IAPAR 81 exhibits moderate tolerance to drought and moderate resistance to anthracnose, rust, powdery mildew, and mosaic virus. On the other hand, it is susceptible to bacterial blight and angular leaf spot. Line LP 97-28 was created based on multiple crossings (LP97-28 = [bz1977-2//IAPAR 14/Carioca 80 Selection]/[BAT93/2/Selection Carioca 99/Great Northern Nebraska/3//Araúna Selection] also conducted by IAPAR, but it has not yet been released. It belongs to the commercial group carioca, upright bush type, indeterminate growth habit (type II), brown-striped cream grains, and average protein content of 27.65%. It shows low tolerance to water deficit [51].

### 4.2. Field Trial Design, Evaluation of Productivity and Morpho Agronomic Characteristics

We evaluated the parents (IAPAR 81 and LP 97-28), 160 recombinant inbred lines from this cross, and seven controls (SCS Guará, Flor Diniz, IPR Juriti, BRS Pérola, IPR Tangará, BRS Talismã, and BAT93) in field conditions during two agricultural years (2014 and 2015) and two environmental conditions, drought stress (DS) and no drought stress (NS). Using this methodology, we could evaluate the performance of the genotypes in a situation with water supply according to the crop requirements (NS). After the flowering period, the water supply was interrupted, simulating drought conditions for ten days (DS).

Field experiments to evaluate the 160 recombinant inbred lines from IAPAR 81 × LP 97-28 cross to drought tolerance were conducted at the Centro Técnico de Irrigação (CTI) from Universidade Estadual de Maringá (UEM) (Maringá, PR, Brazil). The experiment was specifically located at latitude 23°25′ S and longitude 51°57′. The climate is characterized as warm temperate. The annual average rainfall for the local area is estimated to be 1276 mm, and the annual average temperature is 22 °C at an elevation of 515 m above sea level. The soil is classified as dystroferric red latosol (oxisol) with a pH value ranging from 5.7 to 6.5 and clayey soil texture. Soil contents included sand (122.6 g kg^−1^), silt (120.6 g kg^−1^) and clay (756.8 g kg^−1^).

In 2014, plots were composed of two 1.5 m rows spaced 0.5 m apart, with 1 m between plots. The design experiment was a triple 13 × 13 lattice experiment with two repetitions for each DS and NS experiment in 2014. In 2015, the plot design was 2 m rows spaced 0.5 m apart and 1 m between plots with three repetitions for each DS and NS experiment. The design experiment was a triple 13 × 13 lattice experiment with three repetitions for each DS and NS experiment in 2015.

For both experiments, a 15 m border of cultivar IAPAR 81 avoided lateral water movements through the soil between the DS and NS experimental conditions. All experiments were carried out at the end of July to increase the chances of extending the drought season during flowering and grain filling periods. Harvesting in both agricultural years occurred at the beginning of November.

Every three days, a water supply was established for the two environmental conditions (DS and NS) from the sowing data until the 20th day after emergence, allowing soil humidity to achieve field capacity. After the 20th day, irrigation was implemented to support soil water tension between 6 KPa and 50 KPa. The 6 kPa tension corresponds to field capacity humidity, while 50 kPa was applied as a low soil water tension limit recommended for common bean crops. Soil humidity was measured with 12 tensiometers installed in each DS and NS experiment.

At the phenological stage of flowering (R5), two irrigation management approaches were used for DS and NS conditions. For NS (no drought), irrigation was continued until the end of the crop cycle, keeping the soil water tension between 6 KPa and 50 KPa. For the DS (with drought) condition, irrigation was discontinued after the flowering period (R5) for 10 days. The period of ten days of stress for drought began when the soil water tension was 50 KPa. After a 10-day drought stress simulation, the water supply was re-established using the same strategy as the NS experiment. Environmental data, such as rainfall (mm), temperatures (°C) and soil water tension (KPa), were collected by the meteorological station located at Centro Técnico de Irrigação (CTI).

The following agronomic traits were collected: (a) number of days to flowering (NDF)—days between emergence until flowering, when 50% of the plants from the plots exhibited at least one flower; (b) number of days to maturity (NDM)—days between emergence until 90% of the pods lost their green coloration and became too dry; (c) number of days to grain filling (NDG)—calculation of the days between flowering and maturation; (d) grain yield (GY)—a measurement based on each plot at 14% humidity and calculated in kg ha^−1^; (e) seed yield per day (SYD)—refers to the total weight of seeds per plot/number of days to maturity and is estimated in g.m^−2^; (f) 100-seeds weight (SW)—the weight of a plot sample containing 100 seeds expressed in grams; (g) number of pods per plant (NP)—total number of pods per plot divided by the number of plants per plot; (h) number of seeds per pod (NSP)—number of seeds of 10 pods randomly selected within a plot; (i) plant height (PH)—the distance between soil and tip of leaves measured in cm; (j) drought intensity index (DII)—assessed for each experiment as 1-(Xd/Xp), where Xd corresponds to the mean yield of genotypes under DS condition and Xp is the mean yield of genotypes under ND condition.

### 4.3. Phenotypic Data Analysis

The phenotypic data of each trial were analyzed by fitting SELEGEN software [52]. The prediction of genotypic values was performed via BLUP (best linear unbiased predictor) using estimates of variance components of random factors obtained by the restricted maximum likelihood (REML) method. Mixed models were used to analyze the phenotypic data for one location and one harvest season, and the following mathematical model for an incomplete block design was adopted (Model 17 Selegen):

$$y=X_{r}+Z_{g}+W_{b}+e$$

*y* = data vector; *r* = replication effect vector (assumed as fixed) added to the overall mean; *g* = genotypic effect vector (assumed as random); *b* = block effect vector (assumed as random); and *e* = error or residuals value (random). The uppercase letters represent the incidence matrices for the aforementioned effects.

The significance of the effects of the models was estimated using the likelihood ratio test (LRT), thus configuring a deviance analysis (ANADEV). The goodness of fit was obtained based on studies with and without genotype effects and genotypes × environments interaction. Then, the deviance without that effect was subtracted from each deviance of the complete model, comparing it with the chi-square value (*x*^2^) with 1 degree of freedom at 1% and 5% probability [53].

Mathematically, as follows:

$$LRT=-2ln\left(\frac{ML\;of\;reduced\;model}{MLV\;of\;complete\;model\;}\right)$$

*ln* represents the Naperian logarithm, and *ML* is the maximum likelihood estimation.

The analyses fit the effect of each line on the phenotype as fixed and random to obtain the best linear unbiased estimators (BLUEs) and best linear unbiased predictors (BLUPs), respectively. The best linear unbiased estimates (BLUEs) obtained through the REML/BLUP analysis were used to calculate Pearson’s correlation coefficients. Pearson’s correlation between grain yield (GY), seed yield per day (SYD), number of pods per plant (NP), 100-seed weight (SW), plant height (PH), number of days for flowering (NDF), number of days to mature (NDM) and the number of days to grain filling (NDE) were calculated independently for each of the environmental conditions (NS—no drought stress; DS—with drought stress) evaluated in two agricultural years. Significant differences were assumed at 0.05, 0.01 and 0.001 probability levels.

### 4.4. Genotyping of the RILs Using BARCBean6K_3 BeadChip

Total genomic DNA was isolated from the 160 RIL families (F8 generation) and parents (IAPAR 81 × LP 97-28) using the DNeasy plant mini kit (Qiagen, CA, USA) following the manufacturer’s instructions. The DNA was quantified using a 1.5% agarose gel (Agarose SFR, Amresco, IL, USA) with TBE buffer (tris-borate-ethylenediamine tetraacetic acid) and stained with 1 μg mL^−1^ Sybr Green (Sigma-Aldrich, St. Louis, MI, USA).

The DNA samples were screened with 5398 SNP DNA markers on the BARCBean6K_3 Illumina BeadChip by following the Infinium HD assay ultra protocol (Illumina, Inc., San Diego, CA, USA). The BeadChip was imaged using the Illumina beadarray reader to measure fluorescence intensity. Automatic allele calling for each locus was performed with Genome Studio Genotyping Module v1.8.4 software (Illumina, San Diego, CA, USA) and visually inspected. Any errors in allele calling due to improper cluster identification were corrected, resulting in 4633 high-quality SNPs.

Subsequently, after checking for the quality of SNPs with the software GenomeStudio, we used an electronic spreadsheet in Microsoft Excel to perform the filtering process by excluding monomorphic SNPs among the parents IAPAR 81 (tolerant parent) and LP 97-28 (not tolerant parent), with lost data greater than 5%, and with a minimum allele frequency minimum allele frequency less than 30%. In addition, SNP markers were placed on their respective mapping chromosomes and assigned their nomenclature deposited at the National Center for Biotechnology Information advances (NCBI).

### 4.5. Genetic Mapping

SNP data from the 160 recombinant inbred lines from IAPAR 81 × LP 97-28 genotyped with the BARCBean6K_3 BeadChip and previously filtered were used to construct genetic linkage maps for P. vulgaris. Only SNP markers polymorphic between parents IAPAR 81 and LP 97-28 that segregated in the RIL population and exhibited a minimum allelic frequency of 30% were used for the linkage map construction. The genetic distances (cM) for each of the eleven linkage groups of P. vulgaris were calculated using JoinMap 4.1 software [54]. Default settings for the regression mapping algorithm based on the Kosambi map function were used to define linkage order and distances (cM). The genetic distances (cM) were later used to graphically design the linkage groups using MapChat 2.3 [55].

### 4.6. QTL Analysis

Quantitative trait loci (QTL) analysis was performed using the new adjusted genotypic values of the evaluated characteristics obtained through the REML/BLUP analysis for the years 2014 and 2015 and the combination of agricultural years for each of the environmental conditions (NS—no drought stress; DS—with drought stress) evaluated. QTL analysis was conducted using Win Cartographer V2.5-011 [56]. For this study, we used the composite interval mapping method (CIM), model 6. We set the parameters at a window size of 10 cM and a walking speed of 1 cM. The QTL was considered significant based on the 1000-permutation test for each trait in Win QTL Cartographer to determine the LOD threshold at *p* = 0.05, and the peak LOD score was considered the location of the QTL. The proportion of phenotypic variance accounted for by the QTL was determined using the R2 value. The QTLs were named according to the common bean QTL nomenclature guidelines proposed by Miklas and Porch [57]. Finally, using MapChat 2.3 [55], the QTLs were graphically represented in the genetic linkage maps for P. vulgaris developed using the RIL population IAPAR 81 × LP 97-28.

## 5. Conclusions

The evaluation of the RIL population from the IAPAR81 × LP97-28 cross, under two different conditions of water supplementation, no drought stress (NS) and drought stress (DS), showed a reduction in grain yield, yield per day, and the number of pods per plant when plants were submitted to an environment with a reduction in water supplementation. However, the lines derived from the IAPAR81 × LP97-28 cross have shown potential for overcoming water deficiency during the flowering stages, which is one of the most critical phases for cultivating common beans. Using 773 polymorphic SNP markers in the RIL population, and phenotypic data obtained in the DS and NS conditions, we identified consistent QTLs associated with six traits that ensure adaptation of the RILs to water stress in common beans. Thus, a total of 15 QTLs associated with grain yield, seed yield per day, 100-seed weight, number of pods per plant, plant height, number of days for flowering, and number of days for maturation, were identified on the chromosomes Pv01, Pv02, Pv03, Pv07, Pv08, Pv09, Pv10, and Pv11.

## Figures and Tables

**Table 1:** Drought intensity indices for IAPAR 81, LP97-28 and 160 recombinant inbred lines from this cross and controls for the agricultural years 2014 and 2015 in Maringá, PR, Brazil.

Traits	Drought Intensity Indices											
	2014						2015					
	${\underline{x}}_{g}$	${\underline{x}}_{L}$	${\underline{x}}_{P}$	${\underline{x}}_{P1}$	${\underline{x}}_{P2}$	${\underline{x}}_{L+P}$	${\underline{x}}_{g}$	${\underline{x}}_{L}$	${\underline{x}}_{P}$	${\underline{x}}_{P1}$	${\underline{x}}_{P2}$	${\underline{x}}_{L+P}$
GY	0.59	0.59	0.70	0.72	0.65	0.65	0.30	0.29	0.38	0.21	0.50	0.34
SYD	0.41	0.41	0.44	0.40	0.50	0.42	0.35	0.34	0.40	0.26	0.50	0.37
NP	0.30	0.30	0.59	0.64	0.44	0.46	0.22	0.21	0.12	0.04	0.18	0.17
SW	0.06	0.06	0.16	0.27	-	0.11	0.03	0.03	0.13	0.11	0.15	0.08
PH	0.09	0.09	0.11	0.15	0.07	0.10	0.10	0.10	0.06	0.15	-	0.08
NDF	-	-	0.01	-	0.02	-	0.01	0.01	-	-	0.02	-
NDM	0.03	0.03	0.04	0.02	0.06	0.04	-	-	-	-	0.01	-
NDG	0.10	0.10	0.09	0.05	0.13	0.09	-	-	-	-	-	-

${\underline{x}}_{g}$: general average; ${\underline{x}}_{L}$: average of the 160 RILs; ${\underline{x}}_{P}$: average of the parents; ${\underline{x}}_{P1}$: average of IAPAR 81; ${\underline{x}}_{P2}$: average of LP97-28; ${\underline{x}}_{L+P}$: averages of RILs and parents. GY = grain yield; SYD = seed yield per day; NP = number of pods per plant; SW = 100-seed weight; PH = plant height; NDF = number of days for flowering; NDM = number of days to mature; and NDG = number of days to grain filling.

**Table 2:** Traits evaluated in the parents IAPAR 81 and LP97-28, and in 160 RILs derived from this cross for the experiments performed under drought stress (DS) and no-drought stress condition (NS).

Traits	Year	Con.	Parents		RILs			^§^ *Gê*	*PEV*
			IAPAR 81	LP97-28	Average	Interval	LRT (*x*^2^)		
**Yield and Its Components**									
Grain Yield (kg ha^−1^)	2014	NS	3381.50	2048.17	2363.49	2020.68–3581.05	169.22 **	**	31,674.07
		DS	980.23	819.52	897.38	820.89–1068.32	45.05 **		5134.52
	2015	NS	959.61	1207.77	993.92	744.72–1508.50	297.85 **	**	3147.65
		DS	765.77	751.01	754.47	521.96–1621.87	319.35 **		2369.41
Seed Yield per day (g m^2^)	2014	NS	3.64	4.39	3.96	3.64–4.74	18.73 **	**	0.079
		DS	2.63	2.15	2.37	2.155–2.91	53.28 **		0.037
	2015	NS	2.20	2.74	2.27	1.71–3.55	ns	**	0.017
		DS	1.65	1.63	1.60	1.11–3.45	ns		0.013
Number of pods per plant	2014	NS	17.49	10.23	11.41	10.12–18.13	125.40 **	**	0.613
		DS	7.95	7.05	8.03	7.07–10.40	214.06 **		0.180
	2015	NS	8.10	9.46	8.61	7.13–14.11	118.32 **	**	0.464
		DS	7.44	8.88	7.23	5.59–12.67	179.12 **		0.323
Number of seeds per pod	2014	NS	5.58	5.59	5.60	5.53–5.76	ns	ns	0.020
		DS	5.11	5.13	5.13	5.08–5.24	ns		0.019
	2015	NS	5.10	5.11	5.00	4.91–5.17	ns	*	0.040
		DS	4.58	4.55	4.51	4.36–4.80	ns		0.075
100-seed weight (g)	2014	NS	25.53	17.81	19.39	17.84–25.68	374.43 **	**	0.268
		DS	18.97	16.74	17.79	16.77–19.70	150.26 **		0.349
	2015	NS	21.13	19.93	20.91	19.60–23.78	74.88 **	**	0.736
		DS	20.45	19.17	20.28	18.97–22.68	46.98 **		1.110
Plant height (Cm)	2014	NS	56.39	55.42	56.00	54.28–59.87	26.98 **	**	3.656
		DS	49.80	50.13	50.23	49.31–52.11	8.26 **		2.371
	2015	NS	51.75	49.87	50.49	48.71–53.47	10.51 **	**	6.116
		DS	45.78	47.37	45.65	44.02–49.09	8.00 **		6.347
**Phenology**									
Number of days for flowering	2014	NS	48.02	48.87	48.15	47.59–48.15	92.33 **	**	0.168
		DS	48.02	48.03	48.21	47.92–49.02	34.46 **		0.130
	2015	NS	48.89	9.22	49.00	48.80–49.24	ns	**	0.241
		DS	48.71	48.77	48.64	48.39–48.89	5.87 *		0.196
Number of days to maturity	2014	NS	79.00	80.98	79.67	78.93–80.90	37.3 **	**	0.612
		DS	76.70	78.11	77.67	76.25–80.01	141.25 **		0.641
	2015	NS	87.95	89.31	88.22	87.23–91.16	11.10 **	**	1.640
		DS	94.42	94.40	94.43	94.40–94.46	ns		0.153
Number of days to grain filling	2014	NS	31.36	32.17	31.72	31.33–32.45	10.03 **	**	0.376
		DS	28.84	29.74	29.39	28.29–31.51	73.86 **		0.620
	2015	NS	39.28	39.27	39.04	38.41–41.01	3.87 *	**	1.307
		DS	46.03	46.01	46.05	46.01–46.09	ns		0.147

* and ** = significant by the chi-square test with one degree of freedom: at 5% (3.84) and 1% (6.63), respectively; ns = not significantly; *PEV* = variance of the error of prediction for genotypic values; Con = condition; RIL = recombinant inbred line; ^§^ Gê = interaction between genotype treatments and stress condition.

**Table 3:** Pearson’s correlation coefficients between the average values of the characteristics evaluated in the RIL population IAPAR 81 × LP97-28 cultivated under drought stress (DS) and no-drought stress (NS) conditions in Maringá, PR in the years 2014 and 2015. Values above the diagonal are the correlation coefficients between the traits under drought stress (DS); values below the diagonal are the correlation coefficients between traits no-drought stress (NS).

Traits	GY	SYD	NP	SW	PH	NDF	NDM	NDG
GY		-0.07 ^ns^	0.00 ^ns^	0.05 ^ns^	0.07 ^ns^	−0.04 ^ns^	0.00 ^ns^	−0.01 ^ns^
SYD	0.73 ***		0.72 ***	0.46 ***	0.14 ^ns^	−0.28 ***	−0.34 **	−0.27 ***
NP	0.65 ***	0.57 ***		0.21 **	0.11 ^ns^	−0.11 ^ns^	−0.21 **	−0.21 **
SW	0.42 ***	0.35 ***	0.29 ***		0.18 *	−0.25 **	−0.21 **	−0.13 ^ns^
PH	0.09 ^ns^	0.08 ^ns^	0.02 ^ns^	0.14 ^ns^		−0.07 ^ns^	−0.04 ^ns^	−0.01 ^ns^
NDF	−0.16 *	−0.15 *	−0.13 ^ns^	−0.21 **	0.00 ^ns^		0.04 ***	0.07 ^ns^
NDM	−0.01 ^ns^	−0.07 ^ns^	0.03 ^ns^	−0.20 *	0.09 ^ns^	0.37 ***		0.93 **
NDE	0.08 ^ns^	0.04 ^ns^	0.05 ^ns^	−0.10 ^ns^	0.08 ^ns^	−0.14 ^ns^	0.84 ***	

*, ** and *** = significance at 0.05, 0.01 and 0.001 probability levels, respectively; ^ns^ = not significant; GY = grain yield; SYD = seed yield per day; NP = number of pods per plant; SW = 100-seed weight; PH = plant height; NDF = number of days for flowering; NDM = number of days to mature; and NDG = number of days to grain filling.

**Table 4:** Number of SNP markers, chromosome size and average distance between markers for the 11 common bean chromosomes for the RIL population derived from the IAPAR 81 × LP97-28 cross genotyped with the BARCBean6K_3 BeadChip.

Chromosome	Number of SNP Markers	Chromosome Size	Average Distance between Markers
		––––––––––––––– cM ––––––––––––––	
Pv01	43	117.4	2.73
Pv02	195	101.4	0.52
Pv03	61	84.1	1.38
Pv04	23	13.1	0.57
Pv05	31	71.1	2.29
Pv06	84	95.3	1.13
Pv07	72	91.3	1.27
Pv08	132	66.4	0.50
Pv09	49	61.3	1.25
Pv10	60	66.6	1.11
Pv11	23	47.8	2.07
Total	773	815.8	
Average			1.34

**Table 5:** Description of the quantitative trait loci (QTL) for drought tolerance identified in the common bean RIL population IAPAR 81 × LP97-28 (IL) cultivated under drought stress (DS) and no-drought stress (NS) conditions in Maringá, PR in the agricultural years of 2014 and 2015.

^§^ Traits/QTL	Year	Cond. *	Chr	Peak of QTL Position	QTL INTERVAL	Nearest SNP Marker	^#^ LOD Score	LOD Threshold	^¥^ Add	^¶^ R^2^
				––––––––cM––––––––––––						%
Grain yield										
GY9^IL^	2014	NS	9	59.1	46.7–59.9	ss715640302	3.13	3.1	−136.67	8.7
Yield per day										
SYD7^L^	2014	DS	7	52.1	49.8–57.1	ss715640487	3.83		0.61	12.1
SYD8^IL^	Combined	DS	8	43.0	28.1–49.2	ss715648231	3.53	2.99	−0.56	9.0
SYD9^IL^	2014	NS	9	22.5	13.5–27.3	ss715649357	3.17		0.34	8.3
100-seed weight										
SW7^IL^	2014	DS	7	61.8	57.8–63.3	ss715639385	7.3	2.99	−0.61	15.4
	2015	NS	7	61.0	50.6–64.1	ss715647728	4.54		−0.72	11.2
	2015	DS	7	61.0	57.3–66.1	ss715647728	3.13		−0.61	6.9
	Combined	NS	7	61.0	48.1–61.4	ss715647728	4.25	3.33	−0.54	10.2
	Combined	DS	7	61.0	58.0–61.4	ss715647728	6.42		−0.62	13.8
SW8^IL^	2014	DS	8	3.6	2.1–5.1	ss715649604	9.44	4.67	−0.73	20.6
	2014	DS	8	6.4	5.1–9.5	ss715648220	9.25	4.02	−0.75	21.0
	2015	NS	8	5.1	0.6–5.4	ss715648549	4.49	3.42	−0.75	10.5
	2015	NS	8	7.1	5.4–11.9	ss715648929	4.87	3.6	−0.78	11.3
	2015	DS	8	4.4	1.9–5.1	ss715648219	3.98		−0.74	9.4
	2015	DS	8	11.1	6.5–16.5	ss715648929	6.33	3.92	−0.97	16.6
	Combined	NS	8	2.0	0.0–3.6	ss715650783	3.33		−0.5	8.5
	Combined	NS	8	7.1	5.4–12.1	ss715648929	3.7		−0.52	8.7
	Combined	NS	8	0.0	0.0–1.6	ss715648043	3.08		−0.13	7.5
	Combined	DS	8	4.4	2.7–5.1	ss715648219	8.65	4.87	−0.75	19.3
	Combined	DS	8	6.4	5.1–12.2	ss715648220	8.96	4.67	−0.78	20.5
	Combined	DS	8	15.3	14.3–16.4	ss715649378	6.89		−0.71	17.2
Number of pods per plant										
NP11^IL^	2014	DS	11	38.6	31.1–41.4	ss715647465	4.88	2.65	0.71	13.0
	2015	DS	11	38.6	31.1–41.4	ss715647465	4.88		0.71	13.0
Plant height										
PH1^IL^	2014	DS	1	67.0	61.0–79.7	ss715646868	3.15		−1.48	8.9
	2015	DS	1	67.0	61.0–79.7	ss715646868	3.15		−1.48	8.9
	Combined	DS	1	68.0	61.0–78.3	ss715646868	4.34	2.93	−1.02	11.2
PH2.1^IL^	Combined	NS	2	19.4	18.5–29.8	ss715640941	3.48		−1.26	8.7
PH2.2^IL^	2014	DS	2	96.7	93.8–98.8	ss715646144	3.95	3.69	−1.53	9.8
	2014	DS	2	99.2	98.8–100.2	ss715646929	3.51	3.24	−1.46	8.8
	2015	DS	2	96.7	93.8–98.8	ss715646144	3.95	3.6	−1.53	9.8
	2015	DS	2	99.2	98.8–100.2	ss715646929	3.51	3.24	−1.46	8.8
	Combined	NS	2	98.1	90.4–100.2	ss715645186	3.45		−0.95	8.5
	Combined	DS	2	96.7	94.9–98.8	ss715646144	4.18	3.19	−0.92	9.5
PH3^IL^	Combined	DS	3	66.4	60.7–70.4	ss715650580	4.32	4.54	0.95	9.5
Number of days for flowering										
NDF2^IL^	2014	NS	2	39.7	29.0–42.8	ss715648819	5.55	3.07	0.34	14.1
	2014	NS	2	48.4	43.5–52.7	ss715647526	4.26	3.17	0.3	10.2
	Combined	NS	2	28.8	22.9–35.8	ss715647652	3.51		0.29	13.1
NDF3^IL^	2014	NS	3	48.4	42.4–50.5	ss715647689	5.02		−0.32	12.3
NDF10^IL^	Combined	DS	10	0.0	0.0–7.0	ss715639783	3.38	2.88	0.17	8.6
Number of days to maturity										
NDM7^IL^	2014	DS	7	54.11	50.6–57.2	ss715640487	3.74		0.66	12.1
	2015	NS	7	60.31	55–65.3	ss715647734	4.86	3.38	0.96	12.5
	2015	NS	7	73.41	71.2–74.5	ss715648553	3.86		0.87	10.3
	Combined	NS	7	59.31	50.8–64.2	ss715648619	4.73	3.34	0.53	13.2
	Combined	DS	7	61.81	60.3–65.9	ss715639385	3.17		0.53	8.4

* DS—drought stress condition; NS—no-drought stress condition; Chr—chromosome; ^#^ LOD, odds log. LOD thresholds were calculated by the performance of 1000 permutations with *p* = 0.05; ^¥^ effect of replacing a single allele from one parent to another. Positive values indicate allele of origin for IAPAR 81 and negative for LP97-28; ^¶^ portion of the phenotypic variance explained by the QTL LOD peak using Win Cartographer’s composite interval mapping; ^§^ common bean QTL nomenclature guidelines (http://bic.css.msu.edu/_pdf/Guidelines_QTL_Nomenclature.pdf (accessed on 1 December 2017)).

## Data Availability

All data are presented within the article.
